# Pelvic Vein Obstruction in Chronic Thromboembolic Pulmonary Hypertension: A Novel Association

**DOI:** 10.3390/jcm13061553

**Published:** 2024-03-08

**Authors:** Anjali Vaidya, Anika Vaidy, Mohamad Al-Otaibi, Brooke Zlotshewer, Estefania Oliveros, Huaqing Zhao, Ahmed Sadek, Vladimir Lakhter, Paul R. Forfia, Riyaz Bashir

**Affiliations:** 1 Pulmonary Hypertension, Right Heart Failure, and CTEPH Program, Division of Cardiovascular Disease, Lewis Katz School of Medicine, Temple University, Philadelphia, PA 19140, USA; 2 Department of Medicine, Lewis Katz School of Medicine, Temple University, Philadelphia, PA 19140, USA; 3 Department of Clinical Sciences, Temple University Hospital, Philadelphia, PA 19140, USA

**Keywords:** chronic thromboembolic pulmonary hypertension (CTEPH), pelvic vein obstruction (PVO), pulmonary thromboendarterectomy (PTE), pulmonary embolism (PE), deep vein thrombosis (DVT)

## Abstract

**Background:** Pelvic venous obstruction (PVO), defined as greater than 50% stenosis or occlusion of pelvic veins, is a known risk factor for deep vein thrombosis (DVT). DVT is a known risk factor for chronic thromboembolic pulmonary hypertension (CTEPH), but the prevalence of PVO in CTEPH is unknown. **Methods:** This cross-sectional study at Temple University’s tertiary referral center for Pulmonary Hypertension, Right Heart Failure, and CTEPH sought to identify the presence of PVO in patients with CTEPH who underwent cardiac catheterization, pulmonary angiography, and venography. **Results:** A total of 193 CTEPH patients were referred for pulmonary angiography, and among these, 148 underwent venography. PVO was identified in 65 (44%) patients. Lower extremity (LE) DVT was associated with PVO (*p* = 0.004). The severity of pulmonary hypertension was similar with and without PVO (mean pulmonary artery pressure 43.0 ± 10.3 mm Hg vs. 43.8 ± 12.4 mm Hg, *p* = 0.70), as was the need for pulmonary thromboendarterectomy (69.2% vs. 61.4%, *p* = 0.32). **Conclusions:** Pelvic vein obstruction is common and a novel clinical association in patients with CTEPH, particularly in patients with a history of LE DVT. PVO and its role in CTEPH warrants further study, including the potential role of revascularization to mitigate further risk.

## 1. Introduction

Chronic thromboembolic pulmonary hypertension (CTEPH) is a severe form of pulmonary vascular disease resulting from prior pulmonary thromboemboli. It is classified as group IV pulmonary hypertension (PH) by the World Health Organization, designated by pulmonary vascular obstruction. Left untreated, CTEPH can result in significant morbidity and mortality from dyspnea, exertional fatigue, and severe right heart failure [1]. It is estimated that CTEPH occurs in approximately 4% of the patients who survive acute pulmonary embolism [2].

Several risk factors for CTEPH were described. In a prospective study of acute PE, an increased risk of CTEPH was associated with large perfusion defects, young age, recurrent PE, and idiopathic PE [3]. Hematologic factors associated with prothrombotic states were also found to be associated with an increased risk of CTEPH, with particular emphasis on antiphospholipid antibodies and lupus anticoagulants [4]. Additional medical conditions were associated with CTEPH, including ventriculoatrial shunts, chronic inflammatory diseases, a history of splenectomy, hypothyroidism, and malignancy [1].

In recent years, additional pelvic anatomic conditions have been newly described in association with CTEPH. Uterine fibroids, when large, were described in association with patients with significant recurrent PE and CTEPH who underwent PTE. Most had a visualized compression of the pelvic veins by the fibroids either by direct venography or contrast-enhanced CT venography; interestingly, the majority of these patients underwent hysterectomy and did not have further recurrent venous thromboembolism (VTE) [5]. May-Thurner anatomy, a vascular variant characterized by compression of the left common iliac vein by the overriding right iliac artery, is another pelvic anatomic condition found to be associated with CTEPH. The compression may result in intimal hyperplasia with scarring of the iliac vein, stasis of blood flow, and deep vein thrombosis (DVT), which has the potential for acute and recurrent PE, leading to CTEPH [6]. These associations identify a newly described association and potential mechanism of recurrent DVT and VTE leading to CTEPH. Extrinsic compression of pelvic veins, from mass effect created by nonvascular or vascular structures, comprise an important and underrecognized cause of pelvic vein obstruction (PVO) and VTE. Other causes of PVO include intrinsic vascular stenoses of venous structures caused by chronic organized thrombus from prior episodes of DVT. The anatomic levels of these obstructions range from inferior vena cava to common femoral veins [1]. We hypothesized that there is an important potential association between the broader defined category of PVO conditions and CTEPH. Herein, we studied the prevalence of PVO in CTEPH patients seen in a tertiary referral Pulmonary Hypertension, Right Heart Failure, and CTEPH program. 

## 2. Materials and Methods

This is a retrospective chart review at Temple University Hospital’s Pulmonary Hypertension, Right Heart Failure, and CTEPH Program (Philadelphia, PA, USA), including all patients in the cardiac catheterization laboratory with CTEPH between January 2016 and June 2020. Temple is a national CTEPH referral center in the United States. Excluded from our study were patients who did not have an invasive venography, and their baseline characteristics can be found in the Supplemental Data Tables S1 and S2. The Institutional Review Board at Temple University approved this study. No extramural funding supported this research. 

Cardiovascular experts in the Pulmonary Hypertension, Right Heart Failure, and CTEPH Program diagnosed CTEPH based on clinical history, physical examination, transthoracic echocardiogram, ventilation/perfusion (V/Q) scan, computed tomography angiography (CTA) scan, right heart catheterization (RHC), and invasive pulmonary angiogram. To minimize clinical bias, the diagnoses were made by a multidisciplinary CTEPH committee, including medical expertise from PH cardiology, chest radiology, interventional cardiology, and cardiothoracic surgery. All patients referred to our program during the specified time period were reviewed by the CTEPH committee, and those that were deemed clinically appropriate were referred to our cardiac catheterization lab for further diagnostic evaluation. Invasive venography was performed with RHC and pulmonary angiography and was reviewed to determine if PVO was present. PVO was defined as greater than 50% stenosis or occlusion of the pelvic veins with or without thrombosis. Given the ability to utilize intravascular ultrasound (IVUS) if warranted for further vascular characterization and the extensive diagnostic testing coordinated for patients traveling to our national CTEPH referral center, invasive venography was performed easily at the time of right heart catheterization and pulmonary angiography was used over performing additional noninvasive imaging to assess for PVO.

All patients underwent laboratory testing for underlying primary hypercoagulable disorders, including antiphospholipid syndrome, factor V Leiden gene mutation, antithrombin deficiency, prothrombin gene mutation, protein S deficiency, and protein C deficiency. Anticoagulation therapies and clinical histories of dyslipidemia, hypertension, type 2 diabetes mellitus, and tobacco use were also assessed. We also recorded oral contraceptives or hormone replacement therapy. A lower extremity duplex was performed to evaluate lower extremity venous thrombosis. Patients were examined for post-thrombotic syndrome (swelling or signs of venous hypertension) of the lower extremities.

Treatment modalities for CTEPH were assessed, including pulmonary thromboendarterectomy (PTE) or balloon pulmonary angioplasty (BPA). Proximal or distal CTEPH was described based on surgical specimens in PTE or CTA in those who did not undergo PTE. Proximal CTEPH involves the main and lobar pulmonary artery (PA), while distal CTEPH involves the segmental and subsegmental PA. Pelvic venous revascularization with or without stent placement was also assessed. Predictors of PVO were also evaluated. 

STATA^®^ 16 (StataCorp, College Station, TX, USA) was used for statistical analyses. Continuous variables were reported as mean ± standard deviation (SD) and compared using the Two-sample *t* test. Categorical variables were reported as percentages and compared using the Pearson chi-square test. Univariate analysis was performed to identify predictors of PVO; if significant, predictors were tested in a multivariable regression analysis model. A *p*-value less than 0.05 was considered statistically significant. 

## 3. Results

**Figure 1:**
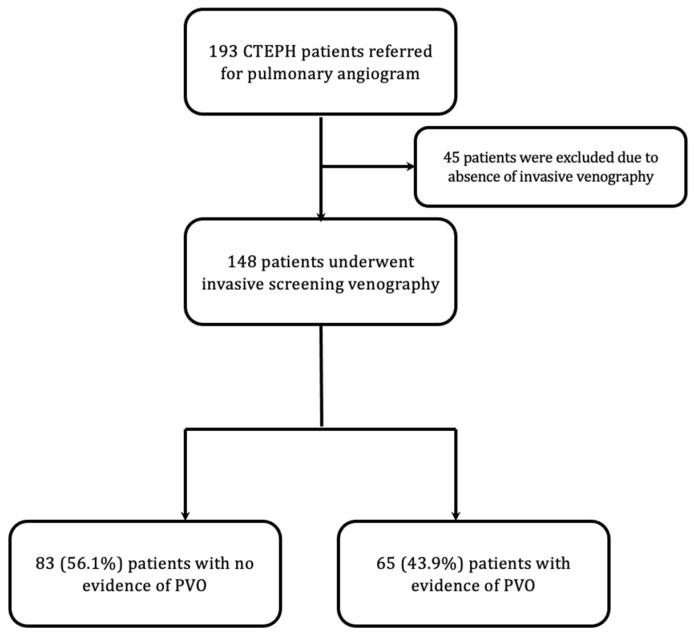
Selection of patients. CTEPH—chronic thromboembolic pulmonary hypertension, PVO—pelvic vein obstruction.

Of the 193 patients referred to our CTEPH program who underwent catheter-based pulmonary angiography, 148 of them had diagnostic invasive lower extremity venography (Figure 1). While it is not broadly standard practice in CTEPH to undergo venography as part of the diagnostic evaluation, many referrals to our CTEPH program with reported idiopathic recurrent VTE were found to be associated with compressive large uterine fibroids and May-Thurner anatomy, leading to an increasing frequency of, and ultimately nearly routine, diagnostic venography at the time of catheter-directed pulmonary angiography to evaluate for PVO. Baseline characteristics are summarized in Table 1. The average age in the entire cohort was 58.8 ± 13.5 years; 53.4% were female, and 46.6% had a hematologic hypercoagulable state. All patients were treated with therapeutic anticoagulation. Sixty-five (44%) patients had PVO (Figure 1). Of those patients with PVO, the average age was 59.4 ± 12.7 years, and 53.8% were female. Those with PVO had a lower body mass index (BMI) (29.1 ± 7.2 vs. 31.7 ± 8.9, *p* = 0.05). There were no significant differences in age, sex, brain type natriuretic peptide (BNP), smoking history, hormonal replacement or contraception use, or hematologic or cardiovascular comorbidities (hypertension, diabetes mellitus type 2, dyslipidemia) between those with and without PVO. As expected, those who had PVO had a greater prevalence of lower extremity DVT (70.8% vs. 47.0%, *p* = 0.004). The baseline characteristics and hemodynamics of patients who did not undergo invasive venography are summarized in Supplemental Tables S1 and S2.

Regarding the anatomic characterization and therapeutic interventions for CTEPH (Table 2), there were no significant differences between those with and without PVO. Proximal CTEPH was found in 50.8% of those with PVO, 54.9% of those without PVO, and 53.1% overall (*p* = 0.620). In patients with PVO, 69.2% underwent PTE, and 16.9% underwent BPA, compared to PTE in 61.4% of those without PVO (*p* = 0.325) and BPA in 24.1% of those without PVO (*p* = 0.287). Thus, while PVO suggests a potential association with large caliber DVT and PE (compared to other known associated causes of smaller caliber VTE in CTEPH such as pacemaker leads, intravascular venous ports, small vein DVT, or red blood cell dyscrasias), this did not correlate with a higher likelihood of proximal CTEPH warranting PTE. 

**Figure 2:**
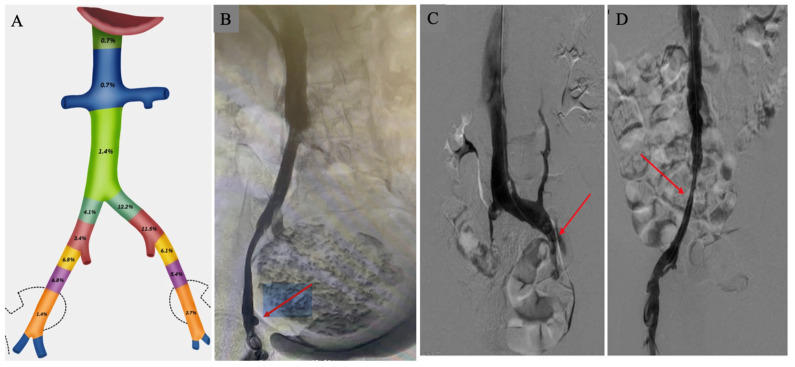
Pelvic vein obstruction. (**A**) Anatomic levels of PVO from inferior vena cava, common iliac veins, external iliac, and internal iliac veins. (**B**) Right external iliac vein compression by large uterine fibroid (arrow). (**C**) Left external iliac vein compression (arrow). (**D**) Right common iliac vein compression (arrow). PVO—pelvic vein obstruction.

The most common anatomic level of PVO was the common iliac veins (31.2%), with the majority involving the left common iliac vein (23.7%). PVO involvement of the external iliac veins was similar between the right (15%) and left (14.2%) sides. Very few PVO occurred at the level of the inferior vena cava (2.8%) (Figure 2). There was no significant difference noted in right (40.0% in those with PVO vs. 37.3% in those without PVO, *p* = 0.742) or left (52.3% in those with PVO vs. 38.6% in those without PVO, *p* = 0.09) lower extremity post-thrombotic syndrome in the two groups. There were no differences in a history of inferior vena cava (IVC) filter placement (30.8% in those with PVO vs. 30.1% in those without PVO, *p* = 0.932) or IVC filter present at the time of the venogram (27.7% in those with PVO vs. 30.5% in those without PVO, *p* = 0.711). Of the 148 total patients, 17 (11.5%) underwent venous stent placement. All of these patients had PVO (*p* < 0.0001) (Table 2). 

In a univariate analysis, BMI < 30 (OR 0.443; 95% CI 0.213–0.923; *p* = 0.0296) and lower extremity DVT (odds ratio [OR]: 3.533; 95% CI 1.58–7.9; *p* = 0.0021) were found to be independent predictors of PVO. With multivariate regression, *p* = 0.031. However, in view of the sample size, the baseline characteristics of the whole cohort without any regression analysis have *p* = 0.05, which may be related to weak effect size as the OR in the multivariate analysis was only 0.91. 

The hemodynamic and procedural characteristics are described in Table 3. Severe pulmonary hypertension was noted in the cohort, with average pulmonary artery systolic pressure 74.1 ± 21.5 mm Hg, pulmonary artery diastolic pressure 25.3 ± 7.8 mm Hg, mean pulmonary artery pressure 43.4 ± 11.5 mm Hg, pulmonary artery wedge pressure 12.2 ± 4.1 mm Hg, and pulmonary vascular resistance (PVR) 8.0 ± 4.4 Wood units. Cardiac output was 4.4 ± 1.2 L per minute (L/min), cardiac index 2.2 ± 0.5 L/min/m^2^, and systemic vascular resistance 1546.9 ± 552.5 dynes/s/cm^−5^. There were no significant differences in hemodynamic parameters, fluoroscopy time (12.6 ± 8.5 min), or amount of iodinated contrast (167.8 ± 63.4 mL) used between patients with or without PVO. 

## 4. Discussion

We report, in this study, a high prevalence (44%) of PVO in CTEPH patients at the tertiary referral Pulmonary Hypertension, Right Heart Failure, and CTEPH Program at Temple University Hospital that underwent diagnostic right heart catheterization, catheter-based pulmonary angiography, and venography. This high rate of prevalence of PVO in CTEPH patients was not previously described. This program previously reported the association of large uterine fibroids with CTEPH and a high prevalence of May-Thurner anatomy (26%) in CTEPH [5,6]. Both prior reports were novel in describing an association between CTEPH and extrinsic anatomic compression of pelvic veins, leading to stasis, venous intimal injury, and risk for lower extremity DVT. While causation has not been proven, both prior reports describe a strong association, which could be a potential mechanism for recurrent PE and CTEPH. This study importantly expands upon this notion, including not just specific etiologies of extrinsic venous compression from uterine fibroids or May-Thurner anatomy but encompassing all anatomic forms of PVO, including extrinsic compression from any cause to intrinsic stenosis of venous structures, ranging from the IVC to the femoral veins.

In this study of CTEPH patients, the PVO by invasive venography was noted at the level of the common iliac veins in one-third of the patients. Importantly, the overwhelming majority of these involved the left common iliac vein, consistent with May-Thurner anatomy. The external iliac veins’ obstruction was equally common in 29.2%, albeit with equal distribution between the right and left sides. This suggests that while the mechanism of PVO in the common iliac veins can be largely attributable to a known congenital anatomic variant, there is a significant prevalence of external iliac vein PVO, which may be comprised of acquired (like uterine fibroids) or congenital abnormalities (like overlying arterial compression). Interestingly, while 30% of our patients had a history of an IVC filter, very few (2.8%) were noted to have obstruction or compression at the level of the IVC.

There were no differences between the groups with and without PVO with regard to age, sex, cardiovascular comorbidities, or tobacco use. Not surprisingly, those with PVO had a 1.5 times greater chance of having a lower extremity DVT and were more likely to undergo venous stenting, a clinical decision made by the interventional cardiologist with vascular medicine expertise, based on the presence of post-thrombotic syndrome in the affected lower extremity. The utility of and threshold for iliac vein stenting remains an important ongoing clinical area of research in pelvic venous disorder management [7,8,9]. The presence of PVO was not associated with a difference in hemodynamic severity of the PH, proximal or distal nature of CTEPH, or candidacy for PTE vs. BPA. The lack of sex-based differences in PVO is very interesting, as we expected this to be higher in females because of extrinsic compression from fibroids and other pelvic masses. Interestingly, in addition to lower extremity DVT, lower BMI was an independent predictor of PVO. High BMI is a known risk factor for lower extremity DVT, even in the absence of anatomic obstruction. Therefore, if a patient with a lower or normal BMI develops DVT, they may be more likely to have an anatomic obstruction like PVO. This was seen previously with May-Thurner anatomy.

In the last several years, advances in the management of CTEPH have included increased surgical expertise for PTE, medical expertise in diagnostic evaluation and perioperative management, approved and available PH medical therapy for inoperable or recurrent CTEPH, and growing interventional interest and experience in BPA [10,11,12,13]. However, the published literature surrounding clinical risk factors for the development of CTEPH evolved minimally over this time period and largely described the same core categories of associated medical conditions. These include hematologic hypercoagulable states, underlying malignancy, splenectomy, ventriculoatrial shunts, and chronic inflammatory states [14,15].

Idiopathic and recurrent PE are known risk factors for the development of CTEPH; however, the mechanism of recurrence has not been well described, and certainly, anatomical risk factors have not been studied [16,17]. As such, it stands to reason that perhaps at least a partial explanation of idiopathic and recurrent PE in the known association with CTEPH is, in fact, related to anatomic mechanical causes such as uterine fibroids, May-Thurner anatomy, or additional causes of PVO, rather than traditionally recognized systemic medical causes such as hypercoagulable or chronic inflammatory states, obesity, malignancy, or ventriculoatrial shunts. It is known that iliac vein compression at different anatomic locations can cause DVT. Regarding potential causal explanations of CTEPH with PVO, the stasis associated with obstruction, intimal injury of the veins caused by compression, and risk for recurrent DVT serve as mechanistic hypotheses in the pathophysiology of developing CTEPH [18,19,20]. 

The limitations of our study include that it is a single-center experience, which may include selection bias in patients referred to our program. That said, given the establishment of our CTEPH program over the last decade as a high-volume referral center in the United States, there is a growing range of geographic variability within the country and referring physician specialties and practice types represented in our patient population. Our study is also limited in the application of invasive venography, which initially was not our standard practice in all patients. As our program identified an association between uterine fibroids and May-Thurner anatomy in CTEPH, it eventually became our standard practice to perform invasive venography at the time of diagnostic pulmonary angiography. As such, not all patients included in our cohort had venography performed, as our practice gradually evolved over the time frame of this study. Additionally, IVUS, noninvasive magnetic resonance, or CT venography were not consistently used in the diagnostic assessment of PVO. Finally, while this is a newly described and potentially important novel mechanism to explore, this study does not establish PVO as a clear cause of thromboembolic disease that ultimately leads to CTEPH.

## 5. Conclusions

PVO encompasses a broad range of under-diagnosed conditions, which can cause proximal lower extremity DVT. The mechanism of PVO causing DVT is understood to be related to venous injury, intimal hyperplasia, and stasis of flow. DVT is a known precursor to PE and the development of CTEPH, and patients with recurrent VTE are known to have an increased risk of developing CTEPH. This study describes a high prevalence of PVO in CTEPH and a higher rate of lower extremity DVT in patients with PVO. As such, while we have not proven a direct causal mechanism of PVO to CTEPH, it is important to recognize this association, as this can have significant therapeutic and clinical implications. Further studies are warranted to validate and characterize this association of PVO with CTEPH and the potential for revascularization as a strategy to mitigate further recurrent VTE in this high-risk patient population. 

## Figures and Tables

**Table 1:** Baseline characteristics of patients with and without pelvic vein obstruction.

	All Patients (n = 148)	Patients without PVO (n = 83 [56%])	Patients with PVO (n = 65 [44%])	*p* Value
Age (years)	58.8 ± 13.5	58.4 ± 14.1	59.4 ± 12.7	0.67
Female (%)	79 (53.4)	44 (53.0)	35 (53.8)	0.09
Mean BNP (pg/mL)	266.9 ± 464.7	315.9 ± 485.9	207.1 ± 433.8	0.17
Mean BMI (kg/m^2^)	30.6 ± 8.3	31.7 ± 8.9	29.1 ± 7.2	0.05
Hypercoagulable state (%)	69 (46.6.)	35 (42.2)	34 (52.3)	0.220
Smoking history (%)	11 (11.5)	7 (8.4)	10 (15.4)	0.188
Hypertension (%)	72 (48.6)	45 (54.2)	27 (41.5)	0.126
Dyslipidemia (%)	48 (32.4)	26 (31.3)	22 (33.8)	0.745
Diabetes mellitus (%)	29 (19.6)	18 (21.2)	11 (16.9)	0.469
Upper extremity DVT (%)	3 (2.0)	1 (1.2)	2 (3.1)	0.423
Lower extremity DVT (%)	85 (57.4)	39 (47.0)	46 (70.8)	0.004
Anticoagulant therapy				0.377
Rivaroxaban (%)	36 (24.3)	19 (22.9)	17 (26.2)	
Apixaban (%)	45 (30.4)	29 (34.9)	16 (24.6)	
Warfarin (%)	56 (37.8)	27 (32.5)	29 (44.6)	
Enoxaparin (%)	8 (5.4)	5 (6.0)	3 (4.6)	
Dabigatran (%)	2 (1.4)	2 (2.4)	0 (0.0)	

BNP—B-type natriuretic peptide, BMI—body mass index, DVT—deep vein thrombosis.

**Table 2:** CTEPH anatomy, clinical characteristics, and therapies with and without PVO.

	All Patients (n = 148)	Patients without PVO (n = 83)	Patients with PVO (n = 65)	*p* Value
Distal CTEPH (%)	70 (46.9)	37 (45.1)	32 (49.2)	0.620
Proximal CTEPH (%)	78 (53.1)	45 (54.9)	33 (50.8)	0.620
Underwent BPA (%)	31 (20.9)	20 (24.1)	11 (16.9)	0.287
Underwent PTE (%)	96 (64.9)	51 (61.4)	45 (69.2)	0.325
IVUS performed (%)	25 (16.9)	2 (2.4)	23 (35.4)	<0.0001
Venous stent placed (%)	17 (11.5)	0 (0.0)	17 (26.2)	<0.0001
Post-thrombotic syndrome in left lower extremity (%)	66 (44.6)	32 (38.6)	34 (52.3)	0.095
Post-thrombotic syndrome in right lower extremity (%)	57 (38.5)	31 (37.3)	26 (40.0)	0.742
IVC filter history (%)	45 (30.4)	25 (30.1)	20 (30.8)	0.932
IVC filter present during catheterization (%)	43 (29.3)	25 (30.5)	18 (27.7)	0.711

PVO—Pelvic Venous Obstruction, CTEPH—chronic thromboembolic pulmonary hypertension, BPA—balloon pulmonary angioplasty, PTE—pulmonary thromboendarterectomy, IVUS—intravascular ultrasound, IVC—inferior vena cava.

**Table 3:** Hemodynamics and procedure characteristics with and without PVO.

	All Patients	Patients without PVO Mean ± SD	Patients with PVO Mean ± SD	*p* Value
Total population (n)	148	83	65	
Right atrial pressure (mmHg)	9.9 ± 4.2	10.0 ± 4.0	9.8 ± 4.5	0.82
Pulmonary artery systolic pressure (mmHg)	74.1 ± 21.5	75.4 ± 23.5	72.5 ± 18.7	0.43
Pulmonary artery diastolic pressure (mmHg)	25.3 ± 7.8	25.2 ± 7.7	25.3 ± 7.9	0.92
Mean Pulmonary artery pressure (mmHg)	43.4 ± 11.5	43.8 ±12.4	43.0 ± 10.3	0.70
Pulmonary capillary wedge pressure (mmHg)	12.2 ± 4.1	12.4 ± 4.1	12.0 ± 4.1	0.63
Cardiac Output (L/min)	4.4 ± 1.2	4.4 ± 1.2	4.3 ± 1.2	0.82
Cardiac Index (L/min/m^2^)	2.2 ± 0.5	2.2 ± 0.5	2.2 ± 0.6	0.71
Systemic vascular resistance (dynes/s/cm^−5^)	1546.9 ± 552.5	1536.0 ± 478.4	1560.5 ± 636.8	0.80
Pulmonary vascular resistance (Wood units)	8.0 ± 4.4	8.0 ± 4.6	8.1 ± 4.2	0.90
Fluoroscopy time (minutes)	12.6 ± 8.5	11.2 ± 5.9	14.5 ± 10.9	0.034
Iodinated contrast dose (mL)	167.8 ± 63.4	164.2 ± 64.0	172.5. ± 62.8	0.43

## Data Availability

Data available in Supplementary Tables, and additional data may be available upon request.

## Supplementary Materials

The following supporting information can be downloaded at: https://www.mdpi.com/article/10.3390/jcm13061553/s1, Table S1: Baseline characteristics of patients that did not undergo invasive venography, Table S2: Hemodynamic data of patients that did not undergo invasive venography.
