# Supplementary material for: Pelvic Vein Obstruction in Chronic Thromboembolic Pulmonary Hypertension: A Novel Association
Source: J Clin Med. 2024 Mar 8;13(6):1553. doi: 10.3390/jcm13061553 (PMC10970950; doi:10.3390/jcm13061553)
Supplement: Supplementary file 1 [file jcm-13-01553-s001.zip › jcm-2883114-supplementary.pdf]

Supplemental data

Supplemental Table S1 - Baseline characteristics of patients that did not undergo invasive venography

Variable	No Venography group<br>n (%) or Mean ± SD
Total Population	45
Age (mean ± SD, years)	59.8 ±17.1
Female	27 (60.0%)
Mean BMI (kg/m^2)	31.4 ± 8.4
Mean BNP (pg/ml)	287.2±366.4
Hypertension N (%)	20 (44.4%)
Dyslipidemia	11 (24.4%)
Diabetes Mellitus	8 (17.8%)
Smoking history	7 (15.6%)
Hypercoagulable States	10 (22.2%)
Lower Extremity DVT	13 (28.9%)
Upper Extremity DVT	2 (4.4%)
Proximal CTEPH	22 (48.9%)
Distal CTEPH	23 (51.1%)
PTE	32 (71.1%)
BPA	8 (17.8%)
IVUS	1 (2.2%)
Inferior Vena Cava (IVC) filter	4 (8.9%)
IVC filter at time of Catheterization	4 (8.9%)

BMI – Body Mass Index, BNP – B-type Natriuretic Peptide, BPA – Balloon Pulmonary Angioplasty, CTEPH – Chronic Thromboembolic Pulmonary Hypertension, DVT – Deep Vein Thrombosis, IVC – Inferior Vena Cava, IVUS – Intravascular Ultrasound, PTE – Pulmonary Thromboendarterectomy

Supplemental Table S2 - Hemodynamic data of patients that did not undergo invasive venography

Variable	Mean ± SD
Total population	45
Right atrial pressure (mmHg)	10.7 ± 5.3
Pulmonary artery mean pressure (mmHg)	44.5 ± 9.8
Pulmonary capillary wedge pressure (mmHg)	12.1 ± 4.7
Cardiac Output (L/min)	4.3 ± 1.1
Cardiac Index (L/min/m^2)	2.2 ± 0.5
Systemic vascular resistance (dynes/sec/cm^{-5})	1518.3± 599.0
Pulmonary vascular resistance (Wood units)	8.7 ± 3.8
Fluoroscopy time (minutes)	15.9 ± 18.9
Iodinated contrast dose (ml)	151.9±91.0
